# Supplementary figures and images for: Who is missed in a community-based survey: Assessment and implications of biases due to incomplete sampling frame in a community-based serosurvey, Choma and Ndola Districts, Zambia, 2022
Source: PLOS Glob Public Health. 2024 Apr 29;4(4):e0003072. doi: 10.1371/journal.pgph.0003072 (PMC11057754; doi:10.1371/journal.pgph.0003072)

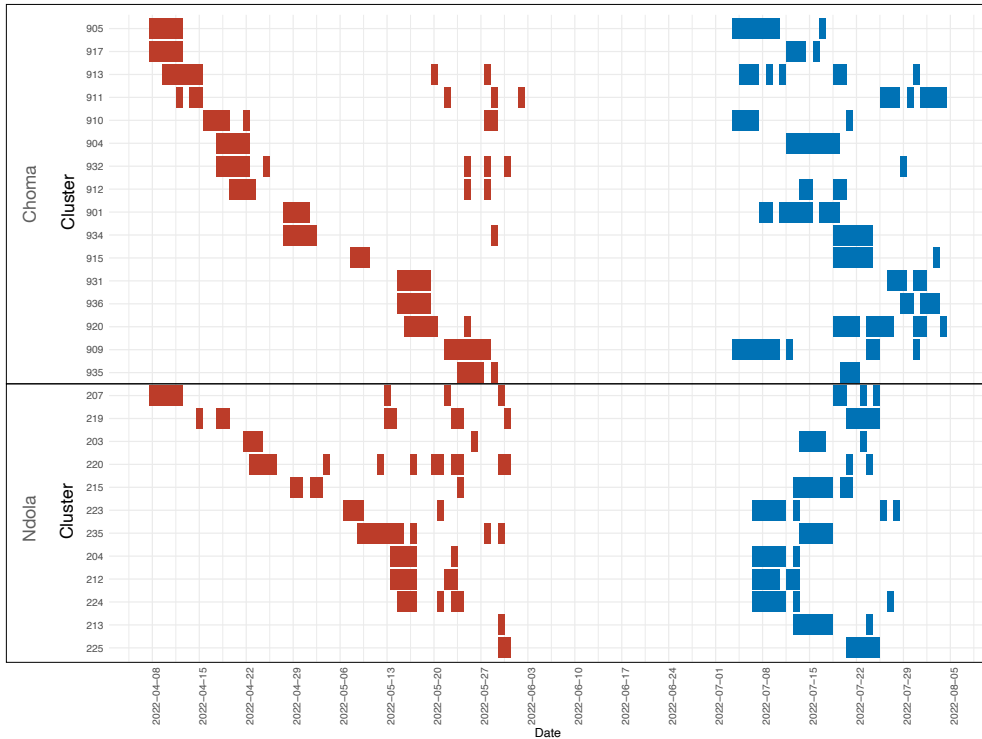

Survey

Original

Missed Population

Supplement: S1 Fig — Gaps in data collection for the same survey indicate that during the initial visit to the cluster, the teams could not locate all households or individuals for enrollment and revisited the cluster later to complete data collection. (PDF) [file pgph.0003072.s002.pdf]

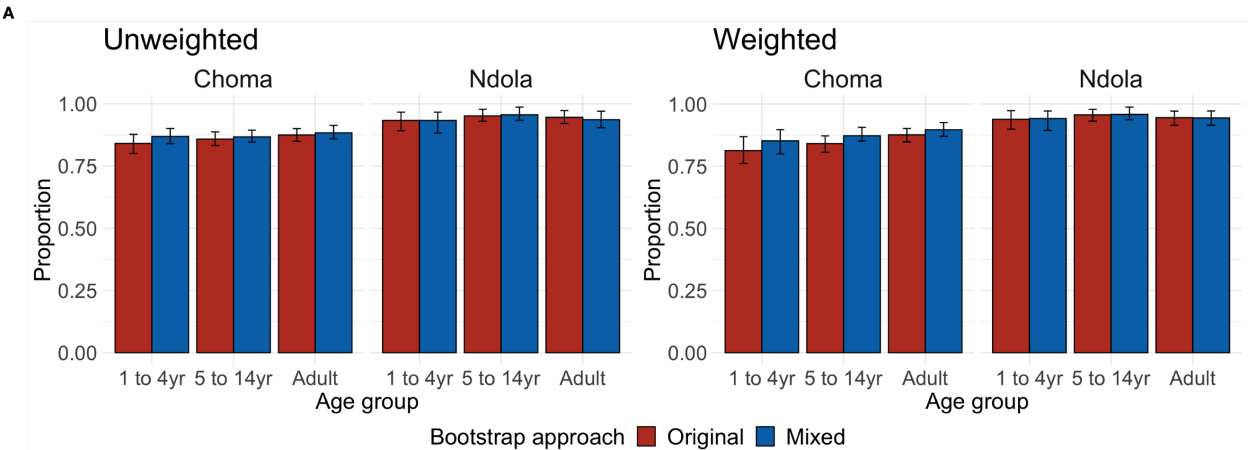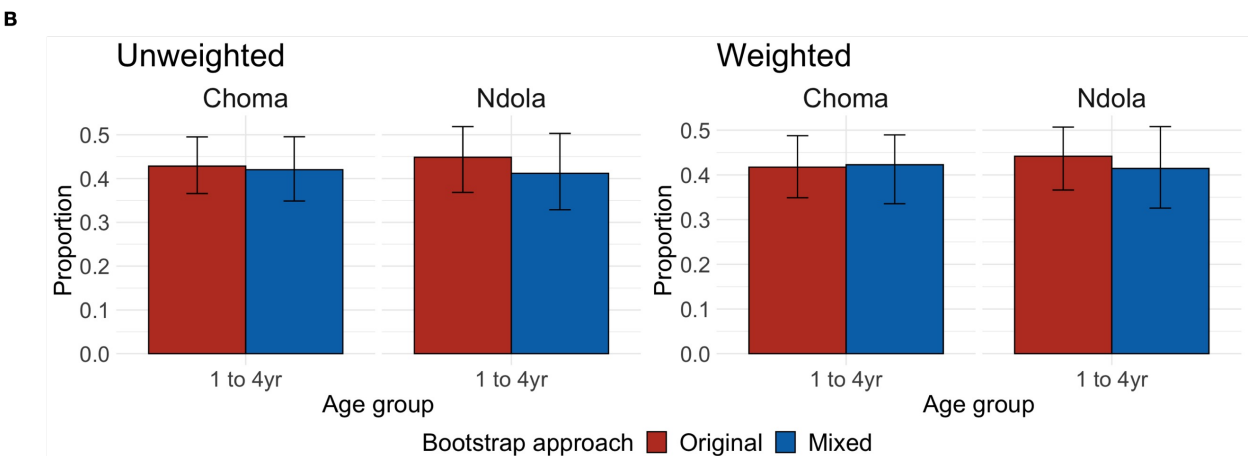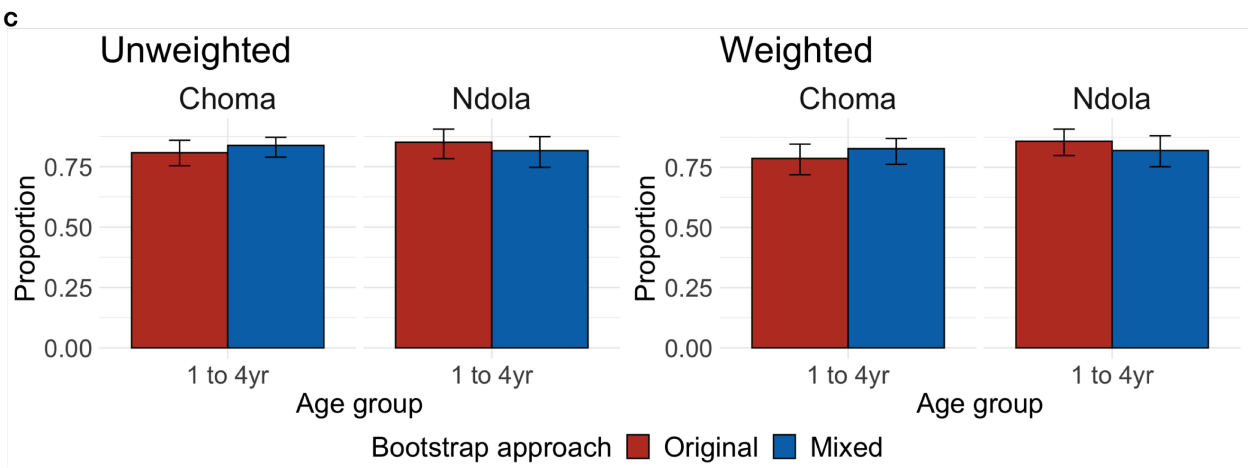

Supplement: S2 Fig — Weighted and unweighted estimates. Weighting was done using the estimated population in each age group in each cluster in the missed population study. A. Health care seeking (actual and theoretical) at facilities of interest (Arthur Davison Children’s Hospital and Choma General Hospital for children 1–4 and 5–14 years old, and Ndola Teaching Hospital and Choma General Hospital for adults 15 years and older). B. MCV2 coverage, children 1–4 years old. C. Measles seroprevalence, children 1–4 years old. (PDF) [file pgph.0003072.s003.pdf]
