# Supplementary material for: Who is missed in a community-based survey: Assessment and implications of biases due to incomplete sampling frame in a community-based serosurvey, Choma and Ndola Districts, Zambia, 2022
Source: PLOS Glob Public Health. 2024 Apr 29;4(4):e0003072. doi: 10.1371/journal.pgph.0003072 (PMC11057754; doi:10.1371/journal.pgph.0003072)
Supplement: S1 Table — (DOCX) [file pgph.0003072.s004.docx]

S1 Table. Number of households selected in each cluster in bootstrapping, by age group and district.

| Age group | Ndola | Choma |
| --- | --- | --- |
| 1 to 4 yr | 10 | 13 |
| 5 to 14 yr | 19 | 24 |
| Adult | 17 | 22 |
