# Supplementary material for: Who is missed in a community-based survey: Assessment and implications of biases due to incomplete sampling frame in a community-based serosurvey, Choma and Ndola Districts, Zambia, 2022
Source: PLOS Glob Public Health. 2024 Apr 29;4(4):e0003072. doi: 10.1371/journal.pgph.0003072 (PMC11057754; doi:10.1371/journal.pgph.0003072)
Supplement: S2 Table — The original serosurvey was carried out in April—June 2022 in Ndola and Choma districts, Zambia, using stratified multi-stage clustering design. The follow-up missed population study was carried out in a subset of clusters of the original survey between July—August 2022. This study was carried out in a subsample of clusters from the original survey; in each selected cluster, a sample of households not available during listing of the original serosurvey, and hence excluded from its sampling frame, were randomly selected. (DOCX) [file pgph.0003072.s005.docx]

S2 Table. Individual demographic characteristics of individuals enrolled in the original study and missed population study, children 1 - 4 years old.

|  | Ndola | | | Choma | | |
| --- | --- | --- | --- | --- | --- | --- |
| Characteristic | Original, N = 101^1^ | Missed Population, N = 106^1^ | p-value^2^ | Original, N = 198^1^ | Missed Population, N = 105^1^ | p-value^2^ |
| Sex |  |  | 0.14 |  |  | 0.24 |
| Female | 59% | 49% |  | 49% | 42% |  |
| Male | 41% | 51% |  | 51% | 58% |  |
| Age | 2 (1) | 2 (1) | 0.38 | 3 (1) | 3 (1) | 0.75 |
| Biological mother alive |  |  | 0.060 |  |  | 0.83 |
| No | 0% | 3.8% |  | 0.5% | 0% |  |
| Yes, in this Household | 88% | 90% |  | 84% | 83% |  |
| Yes, lives elsewhere | 12% | 6.6% |  | 16% | 17% |  |
| Caregiver sex |  |  | >0.99 |  |  | 0.17 |
| Female | 98% | 98% |  | 96% | 99% |  |
| Male | 2.0% | 1.9% |  | 4.0% | 1.0% |  |
| Caregiver age | 34 (10) | 32 (10) | 0.11 | 32 (11) | 32 (11) | 0.63 |
| Wealth score | 1.7 (1.3) | 1.4 (1.1) | **<0.001** | -1.4 (2.7) | -2.0 (2.8) | **<0.001** |
| ^1^%; Mean (SD) | | | | | | |
| ^2^Pearson's Chi-squared test; Wilcoxon rank sum test; Fisher's exact test | | | | | | |
