# Supplementary material for: Who is missed in a community-based survey: Assessment and implications of biases due to incomplete sampling frame in a community-based serosurvey, Choma and Ndola Districts, Zambia, 2022
Source: PLOS Glob Public Health. 2024 Apr 29;4(4):e0003072. doi: 10.1371/journal.pgph.0003072 (PMC11057754; doi:10.1371/journal.pgph.0003072)
Supplement: S3 Table — The original serosurvey was carried out in April—June 2022 in Ndola and Choma districts, Zambia, using stratified multi-stage clustering design. The follow-up missed population study was carried out in a subset of clusters of the original survey between July—August 2022. This study was carried out in a subsample of clusters from the original survey; in each selected cluster, a sample of households not available during listing of the original serosurvey, and hence excluded from its sampling frame, were randomly selected. (DOCX) [file pgph.0003072.s006.docx]

S3 Table. Individual demographic characteristics of individuals enrolled in the original study and missed population study, children 5 – 14 years old.

|  | Ndola | | | Choma | | |
| --- | --- | --- | --- | --- | --- | --- |
| Characteristic | Original, N = 208^1^ | Missed Population, N = 185^1^ | p-value^2^ | Original, N = 371^1^ | Missed Population, N = 162^1^ | p-value^3^ |
| Sex |  |  | 0.28 |  |  | 0.44 |
| Female | 49% | 54% |  | 52% | 48% |  |
| Male | 51% | 46% |  | 48% | 52% |  |
| Age | 9 (3) | 9 (3) | 0.30 | 9 (3) | 9 (3) | 0.97 |
| Biological mother alive |  |  | 0.65 |  |  | 0.22 |
| No | 5.8% | 8.1% |  | 1.3% | 3.1% |  |
| Yes, in this Household | 76% | 74% |  | 67% | 67% |  |
| Yes, lives elsewhere | 18% | 18% |  | 31% | 30% |  |
| Don't Know | 0% | 0% |  | 0% | 0.6% |  |
| Caregiver sex |  |  | 0.35 |  |  | 0.77 |
| Female | 95% | 93% |  | 91% | 91% |  |
| Male | 4.8% | 7.0% |  | 9.4% | 8.6% |  |
| Caregiver age | 38 (11) | 38 (12) | 0.78 | 40 (13) | 38 (13) | 0.060 |
| Wealth score | 2.0 (1.1) | 1.5 (1.1) | **<0.001** | -1.3 (2.7) | -1.6 (2.9) | **0.003** |
| ^1^%; Mean (SD) | | | | | | |
| ^2^Pearson's Chi-squared test; Wilcoxon rank sum test | | | | | | |
| ^3^Pearson's Chi-squared test; Wilcoxon rank sum test; Fisher's exact test | | | | | | |
