# Supplementary material for: Who is missed in a community-based survey: Assessment and implications of biases due to incomplete sampling frame in a community-based serosurvey, Choma and Ndola Districts, Zambia, 2022
Source: PLOS Glob Public Health. 2024 Apr 29;4(4):e0003072. doi: 10.1371/journal.pgph.0003072 (PMC11057754; doi:10.1371/journal.pgph.0003072)
Supplement: S4 Table — The original serosurvey was carried out in April—June 2022 in Ndola and Choma districts, Zambia, using stratified multi-stage clustering design. The follow-up missed population study was carried out in a subset of clusters of the original survey between July—August 2022. This study was carried out in a subsample of clusters from the original survey; in each selected cluster, a sample of households not available during listing of the original serosurvey, and hence excluded from its sampling frame, were randomly selected. (DOCX) [file pgph.0003072.s007.docx]

S4 Table. Individual demographic characteristics of individuals enrolled in the original study and missed population study, adults 15 years and older.

|  | Ndola | | | Choma | | |
| --- | --- | --- | --- | --- | --- | --- |
| Characteristic | Original, N = 186^1^ | Missed Population, N = 367^1^ | p-value^2^ | Original, N = 347^1^ | Missed Population, N = 305^1^ | p-value^2^ |
| Sex |  |  | **<0.001** |  |  | **0.002** |
| Female | 71% | 54% |  | 67% | 55% |  |
| Male | 29% | 46% |  | 33% | 45% |  |
| Age | 35 (15) | 34 (15) | 0.67 | 37 (15) | 36 (16) | 0.28 |
| Household with kids | 81% | 60% | **<0.001** | 83% | 64% | **<0.001** |
| Occupation |  |  | **<0.001** |  |  | **<0.001** |
| Craft and related trades workers | 7.5% | 7.1% |  | 3.7% | 2.3% |  |
| Elementary occupations | 5.9% | 16% |  | 2.0% | 14% |  |
| None / homemaker / retired | 36% | 15% |  | 12% | 6.6% |  |
| Other^3^ | 5.9% | 8.2% |  | 2.3% | 2.0% |  |
| Professional | 9.1% | 9.0% |  | 4.0% | 5.9% |  |
| Pupil/Student | 7.0% | 13% |  | 4.9% | 8.9% |  |
| Service and sales workers | 20% | 26% |  | 18% | 32% |  |
| Skilled agricultural, forestry and fisheries workers | 6.5% | 1.1% |  | 50% | 25% |  |
| Technicians and associate professionals | 1.6% | 4.1% |  | 2.9% | 3.3% |  |
| Wealth | 2.0 (1.1) | 1.3 (1.2) | **<0.001** | -1.4 (2.7) | -1.6 (2.8) | **0.005** |
| ^1^%; Mean (SD) | | | | | | |
| ^2^Pearson's Chi-squared test; Wilcoxon rank sum test | | | | | | |
| ^3^Other category includes armed forces, clerical support workers, managers, and plant machine operators and assemblers | | | | | | |
