# Supplementary material for: Who is missed in a community-based survey: Assessment and implications of biases due to incomplete sampling frame in a community-based serosurvey, Choma and Ndola Districts, Zambia, 2022
Source: PLOS Glob Public Health. 2024 Apr 29;4(4):e0003072. doi: 10.1371/journal.pgph.0003072 (PMC11057754; doi:10.1371/journal.pgph.0003072)
Supplement: S5 Table — The follow-up missed population study was carried out in a subset of clusters of the original survey between July—August 2022. This study was carried out in a subsample of clusters from the original survey; in each selected cluster, a sample of households not available during listing of the original serosurvey, and hence excluded from its sampling frame, were randomly selected. (DOCX) [file pgph.0003072.s008.docx]

S5 Table. Healthcare-seeking behavior reported by adults, stratified by living in households with or without children

|  | Adults in households with kids | | | | | | Adults in households without kids | | | | | |
| --- | --- | --- | --- | --- | --- | --- | --- | --- | --- | --- | --- | --- |
|  | Ndola | | | Choma | | | Ndola | | | Choma | | |
| Characteristic | Original, N = 151^1^ | Missed Population, N = 222^1^ | p-value^2^ | Original, N = 287^1^ | Missed Population, N = 194^1^ | p-value^2^ | Original, N = 35^1^ | Missed Population, N = 145^1^ | p-value^2^ | Original, N = 60^1^ | Missed Population, N = 111^1^ | p-value^2^ |
| *Health care seeking at Choma General Hospital, Arthur Davison Children’s Hospital, and/or Ndola Teaching Hospital* | | | | | | | | | | | | |
| Would visit health facility |  |  | **0.001** | 86% | 97% | **<0.001** | 100% | 99% | >0.99 | 95% | 95% | >0.99 |
| Don't know | 0.7% | 0% |  |  |  |  |  |  |  | 98% | 98% | >0.99 |
| No | 7.9% | 1.4% |  |  |  |  |  |  |  |  |  |  |
| Yes | 91% | 99% |  |  |  |  |  |  |  |  |  |  |
| Way to travel to health facility |  |  | 0.35 |  |  | **0.020** |  |  | **0.017** |  |  | 0.066 |
| Other | 1.3% | 0% |  | 1.0% | 0.5% |  |  |  |  |  |  |  |
| Private motorized transport (e.g., personal car or scooter) | 3.3% | 5.0% |  | 2.4% | 7.7% |  | 20% | 9.0% |  | 1.7% | 12% |  |
| Public motorized transport (e.g., bus, taxi) | 93% | 92% |  | 72% | 61% |  | 74% | 90% |  | 72% | 57% |  |
| Walk | 2.0% | 2.7% |  | 21% | 27% |  | 5.7% | 0.7% |  | 25% | 29% |  |
| Bicycle |  |  |  | 3.8% | 4.1% |  |  |  |  | 1.7% | 2.7% |  |
| Time to travel to health facility |  |  | 0.80 |  |  | **<0.001** |  |  | 0.22 |  |  | 0.84 |
| Less than 30 mins | 11% | 14% |  | 13% | 24% |  | 18% | 11% |  | 33% | 30% |  |
| 30 mins - less than 1 hr | 42% | 39% |  | 14% | 23% |  | 44% | 47% |  | 15% | 22% |  |
| 1 hr - less than 2 hrs | 38% | 41% |  | 24% | 24% |  | 29% | 39% |  | 25% | 25% |  |
| 2 hrs - less than 3 hrs | 7.6% | 5.9% |  | 28% | 13% |  | 8.8% | 2.1% |  | 17% | 13% |  |
| 3 or more hours | 0.7% | 0.5% |  | 21% | 16% |  | 0% | 0.7% |  | 10% | 10% |  |
|  |  |  |  |  |  |  |  |  |  |  |  |  |
| *General healthcare seeking behavior* | | | | | | | | | | | | |
| Money big barrier to health care seeking | 72% | 52% | **<0.001** | 78% | 91% | **<0.001** | 60% | 52% | 0.42 | 78% | 82% | 0.56 |
| Distance big problem to health care seeking | 38% | 18% | **<0.001** | 62% | 54% | 0.10 | 26% | 15% | 0.14 | 50% | 48% | 0.78 |
| ^1^% | | | | | | |  |  |  |  |  |  |
| ^2^Fisher's exact test; Pearson's Chi-squared test | | | | | | |  |  |  |  |  |  |
