# Supplementary material for: Who is missed in a community-based survey: Assessment and implications of biases due to incomplete sampling frame in a community-based serosurvey, Choma and Ndola Districts, Zambia, 2022
Source: PLOS Glob Public Health. 2024 Apr 29;4(4):e0003072. doi: 10.1371/journal.pgph.0003072 (PMC11057754; doi:10.1371/journal.pgph.0003072)
Supplement: S6 Table — The original serosurvey was carried out in April—June 2022 in Ndola and Choma districts, Zambia, using stratified multi-stage clustering design. The follow-up missed population study was carried out in a subset of clusters of the original survey between July—August 2022. This study was carried out in a subsample of clusters from the original survey; in each selected cluster, a sample of households not available during listing of the original serosurvey, and hence excluded from its sampling frame, were randomly selected. (DOCX) [file pgph.0003072.s009.docx]

S6 Table. History of fever / rash symptoms in the past two weeks and diagnosis of suspected measles, children 1 – 4 and 5 – 14 years old

|  | Children 1 – 4 years old | | | | | |  | Children 5 – 14 years old | | | | |  |
| --- | --- | --- | --- | --- | --- | --- | --- | --- | --- | --- | --- | --- | --- |
|  | Ndola | | | Choma | | |  | Ndola | | | Choma | |  |
| Characteristic | Original, N = 101^1^ | Missed Population, N = 106^1^ | p-value^2^ | Original, N = 198^1^ | Missed Population, N = 105^1^ | p-value^2^ |  | Original, N = 208^1^ | Missed Population, N = 185^1^ | p-value^2^ | Original, N = 371^1^ | Missed Population, N = 162^1^ | p-value^2^ |
| Had fever / rash |  |  | 0.53 |  |  | >0.99 |  |  |  | 0.24 |  |  | 0.85 |
| Yes | 5.9% | 3.8% |  | 5.1% | 4.8% |  |  | 4.8% | 2.7% |  | 6.2% | 6.8% |  |
| No | 94% | 96% |  | 95% | 95% |  |  | 95% | 97% |  | 94% | 93% |  |
| Don't know | 0% | 0% |  | 0% | 0% |  |  | 0% | 0.5% |  | 0% | 0% |  |
| Suspected measles (among those with fever + rash) |  |  | 0.13 |  |  | >0.99 |  |  |  | 0.51 |  |  | 0.23 |
| Yes | 0% | 25% |  | 10% | 20% |  |  | 30% | 0% |  | 26% | 0% |  |
| No | 100% | 50% |  | 80% | 80% |  |  | 70% | 100% |  | 70% | 91% |  |
| Did not seek care | 0% | 25% |  | 10% | 0% |  |  | 0% | 0% |  | 4.3% | 9.1% |  |
| ^1^% | | | | | | |  | | | | | |  |
| ^2^Fisher's exact test | | | | | | |  | | | | | |  |
