# Supplementary material for: Who is missed in a community-based survey: Assessment and implications of biases due to incomplete sampling frame in a community-based serosurvey, Choma and Ndola Districts, Zambia, 2022
Source: PLOS Glob Public Health. 2024 Apr 29;4(4):e0003072. doi: 10.1371/journal.pgph.0003072 (PMC11057754; doi:10.1371/journal.pgph.0003072)
Supplement: S7 Table — We used bootstrapping procedure to simulate inclusion of missed households in the sampling frame of a serosurvey carried out in Ndola and Choma districts, Zambia, in April—June 2022. (DOCX) [file pgph.0003072.s010.docx]

**S7 Table.** **Results of unweighted and weighted bootstrapping.** Estimates are proportions of the population estimated to have the outcome of interest. “Original sampling frame” is the scenario where bootstrapping was done using a sampling frame that excluded the missed population households (similar to how the parent study was carried out). “Mixed sampling frame” refers to a sampling frame where households from the original study and the missed population were included. The intervals presented are 2.5^th^ and 97.5^th^ bootstrap intervals for each indicator.

|  |  | Unweighted | | Weighted | |
| --- | --- | --- | --- | --- | --- |
| District | Age group | Original sampling frame | Mixed sampling frame | Original sampling frame | Mixed sampling frame |
| *Would seek care at ADCH / CG / NTH* | | | | | |
| Choma | 1 to 4 yr | 0.841 [0.8; 0.878] | 0.869 [0.84; 0.901] | 0.813 [0.761; 0.868] | 0.852 [0.799; 0.897] |
|  | 5 to 14 yr | 0.858 [0.832; 0.887] | 0.867 [0.846; 0.894] | 0.841 [0.806; 0.872] | 0.872 [0.851; 0.906] |
|  | Adult | 0.875 [0.849; 0.901] | 0.884 [0.859; 0.913] | 0.876 [0.848; 0.902] | 0.896 [0.87; 0.925] |
| Ndola | 1 to 4 yr | 0.933 [0.892; 0.967] | 0.933 [0.883; 0.967] | 0.938 [0.899; 0.973] | 0.942 [0.894; 0.972] |
|  | 5 to 14 yr | 0.952 [0.93; 0.978] | 0.956 [0.934; 0.987] | 0.956 [0.931; 0.978] | 0.958 [0.936; 0.987] |
|  | Adult | 0.946 [0.921; 0.973] | 0.936 [0.904; 0.971] | 0.945 [0.914; 0.971] | 0.944 [0.914; 0.972] |
| *MCV2 Coverage* | | | | | |
| Choma | 1 to 4 yr | 0.429 [0.366; 0.495] | 0.42 [0.349; 0.496] | 0.418 [0.349; 0.488] | 0.423 [0.336; 0.489] |
| Ndola | 1 to 4 yr | 0.449 [0.368; 0.519] | 0.412 [0.329; 0.503] | 0.442 [0.366; 0.507] | 0.415 [0.326; 0.508] |
| *Measles seroprevalence* | | | | | |
| Choma | 1 to 4 yr | 0.808 [0.754; 0.859] | 0.839 [0.791; 0.873] | 0.787 [0.719; 0.846] | 0.828 [0.752; 0.871] |
| Ndola | 1 to 4 yr | 0.851 [0.783; 0.906] | 0.832 [0.756; 0.883] | 0.858 [0.799; 0.909] | 0.826 [0.75; 0.891] |
