# Supplementary material for: Who is missed in a community-based survey: Assessment and implications of biases due to incomplete sampling frame in a community-based serosurvey, Choma and Ndola Districts, Zambia, 2022
Source: PLOS Glob Public Health. 2024 Apr 29;4(4):e0003072. doi: 10.1371/journal.pgph.0003072 (PMC11057754; doi:10.1371/journal.pgph.0003072)
Supplement: S1 Appendix — (DOCX) [file pgph.0003072.s011.docx]

S1 Appendix. Graphical summary of study methods


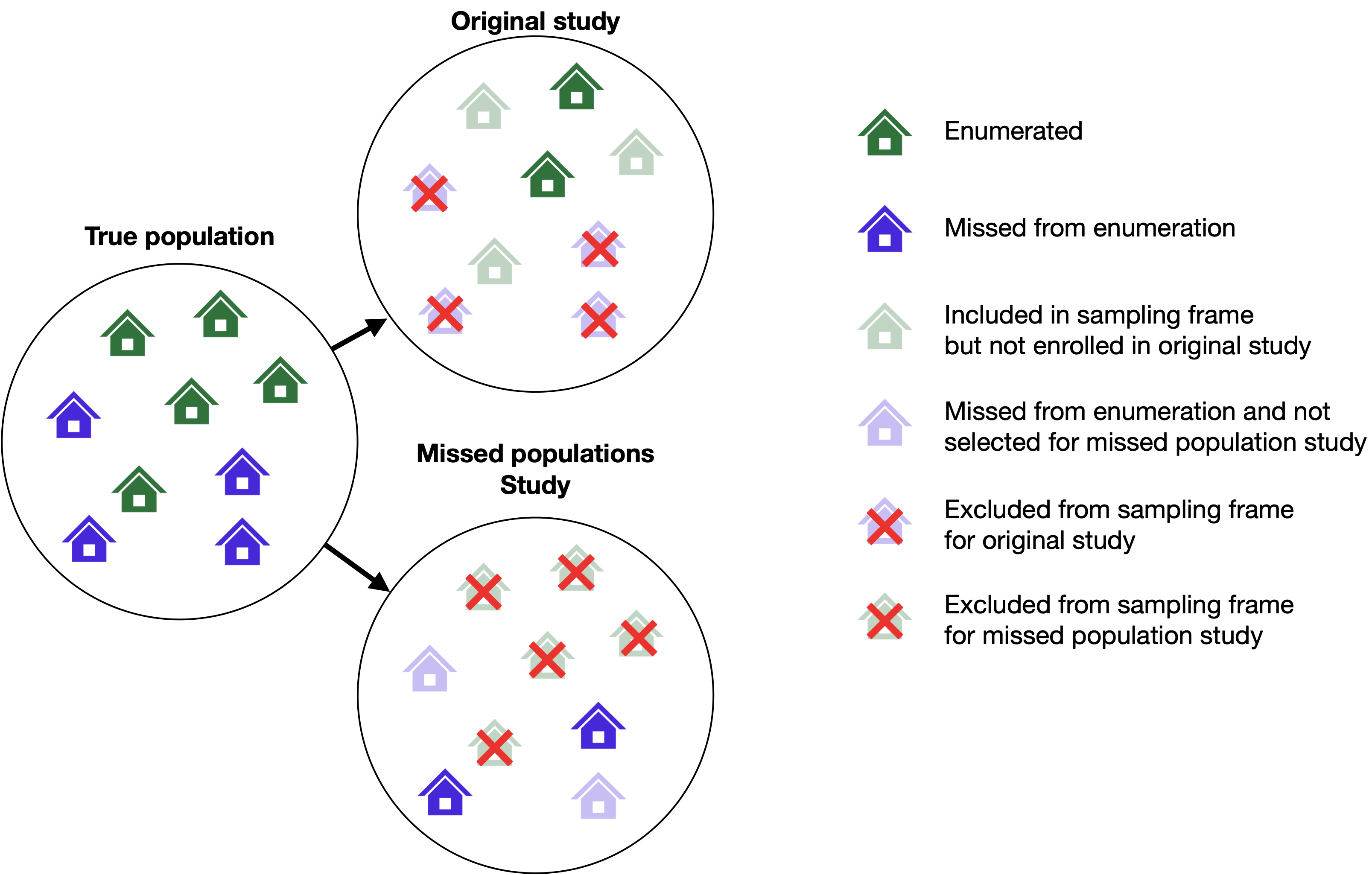

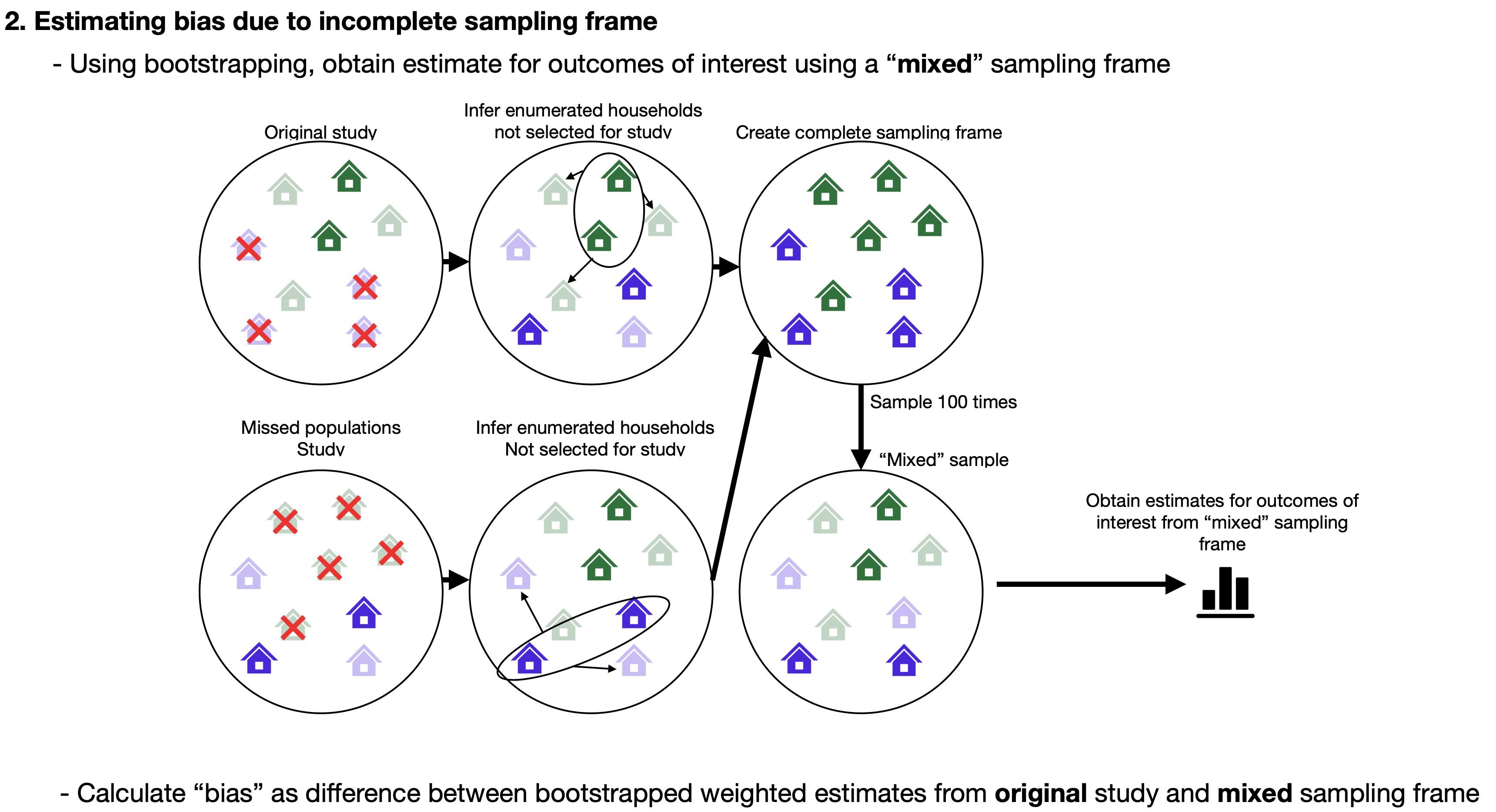


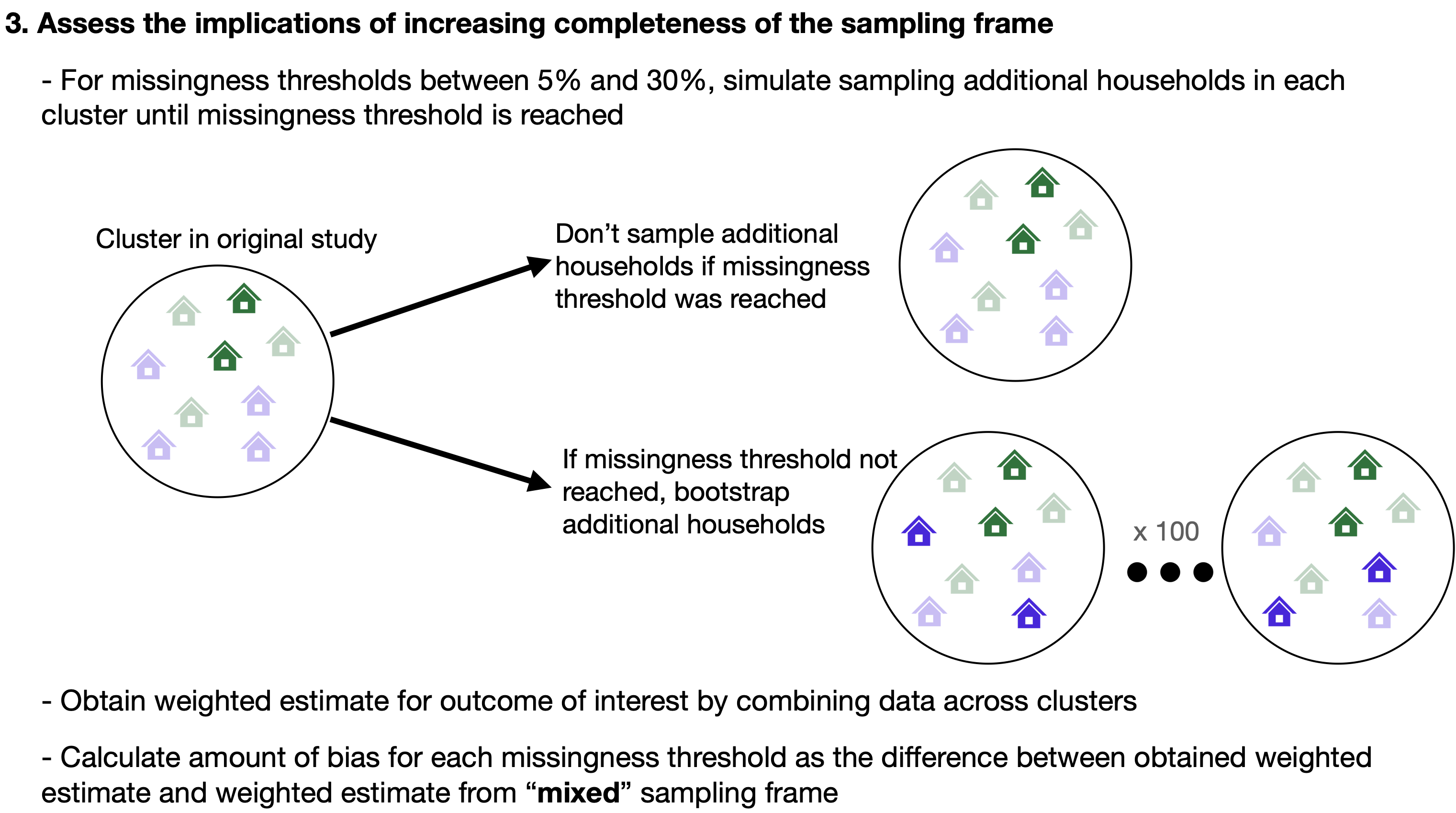


_____________________________

**Graphics attributions:**

Icons were obtained from thenounproject.com, using Royalty-Free License. Under this license, no attribution is required; however, references to the icons are provided below.

**References**

“Bar chart” by mikicon (Royalty-Free License) <https://thenounproject.com/icon/bar-chart-1056801/>

“Big Family” by Gan Khoon Lay (Royalty-Free License) <https://thenounproject.com/icon/big-family-1250862/>

“House” by Aleksandr Vector (Royalty-Free License) <https://thenounproject.com/icon/house-1008968/>

“Medical chart” by Symbolon IT (Royalty-Free License) <https://thenounproject.com/icon/medical-chart-824442/> “Money Bills” by Andy Horvath (Royalty-Free License) <https://thenounproject.com/icon/money-bills-4924987/>

“Question mark” by Phumpsky (Royalty-Free License) <https://thenounproject.com/icon/question-mark-3653884/>
