# Supplementary material for: Who is missed in a community-based survey: Assessment and implications of biases due to incomplete sampling frame in a community-based serosurvey, Choma and Ndola Districts, Zambia, 2022
Source: PLOS Glob Public Health. 2024 Apr 29;4(4):e0003072. doi: 10.1371/journal.pgph.0003072 (PMC11057754; doi:10.1371/journal.pgph.0003072)
Supplement: S2 Appendix — (DOCX) [file pgph.0003072.s012.docx]

S2 Appendix. Definitions of outcome and explanatory variables

**The facility of interest**: Facilities of interest were selected as referral-level facilities within the two districts, where individuals may be referred for care from primary-level facilities like health centers. In Choma District, for all age groups, this was Choma General Hospital. In Ndola District, for children 1 – 4 years old and 5 – 14 years old, the facility of interest was Arthur Davison Children’s Hospital. For adults, this was Ndola Teaching Hospital. Note that these were also the health facilities where residual specimens were collected for assessing age-specific measles seroprevalence in Ndola and Choma districts; the original survey was carried out as validation of the facility-based seroprevalence estimates.

**Healthcare utilization:** Healthcare utilization was measured as having visited the health facility of interest for those previously referred or intent to visit the facility of interest if theoretically referred for those who had never been referred.

**Occupations**: Occupations were coded according to major occupation groups defined by the International Labour Office’s International Standard Classification of Occupations (1). Data on respondents’ occupations were collected as a free-response question and then coded into ten major groups: managers; professionals; technical and associate professionals; clerical support workers; services and sales workers; skilled agricultural, forestry and fishery workers; craft and related trades workers; plant and machine operators, and assemblers; elementary occupations; and armed forces occupations. Subgroups in each of these major groups included the following:

Managers: Chief executives, senior officials and legislators; administrative and commercial managers; production and specialized services managers; hospitality, retail and other services managers.

Professionals: science and engineering professionals; health professionals; teaching professionals; business and administration professionals; information and communications technology professionals; legal, social and cultural professionals.

Technicians and associate professionals: science and engineering associate professionals; health associate professionals; business and administration associate professionals; legal, social, cultural and related associate professionals; information and communications technicians.

Clerical support workers: general and keyboard clerks; customer services clerks; numerical and material recording clerks; other clerical support workers.

Services and sales workers: personal services workers; sales workers; personal care workers; protective services workers.

Skilled agricultural, forestry and fishery workers: market-oriented skilled agricultural workers; market-oriented skilled forestry, fishery and hunting workers; subsistence farmers, fishers, hunters and gatherers.

Craft and related trades workers: building and related trades workers (excluding electricians); metal, machinery and related trades workers; handicraft and printing workers; electrical and electronic trades workers; food processing, woodwork, garment and other craft and related trades workers.

Plant and machine operators and assemblers: Stationary plant and machine operators; assemblers; drivers and mobile plant operators

Elementary occupations: cleaners and helpers; agricultural, forestry and fishery labourers; labourers in mining, construction, manufacturing and transport; food preparation assistants; street and related sales and services workers; refuse workers and other elementary workers

Armed forces occupations: commissioned armed forces officers; non-commissioned armed forces officers; armed forces occupations, other ranks.

A full description of definitions of each major and sub-major group is provided elsewhere (1).
