# Supplementary material for: Who is missed in a community-based survey: Assessment and implications of biases due to incomplete sampling frame in a community-based serosurvey, Choma and Ndola Districts, Zambia, 2022
Source: PLOS Glob Public Health. 2024 Apr 29;4(4):e0003072. doi: 10.1371/journal.pgph.0003072 (PMC11057754; doi:10.1371/journal.pgph.0003072)
Supplement: S3 Appendix — (DOCX) [file pgph.0003072.s013.docx]

S3 Appendix. Multivariable mixed-effects model

We fit a logistic model independently for each age group. The outcome was a binary variable, defined as 1 if the individual was included in missed population dataset and 0 if they were included in the original study. The mixed effects model had cluster as a random effect and the following fixed effects: district (Choma or Ndola), sex (male or female), sex of head of household (male or female), wealth score, number of people in households, and whether money was a big problem for seeking healthcare (yes or no). For children 1 to 4 years old, we also included a DPT2 vaccine variable (three levels: yes, documented; yes, recall; and no).

|  | Children 1 - 4 years old | | Children 5 – 14 years old | | Adults | |
| --- | --- | --- | --- | --- | --- | --- |
| Characteristic | OR [95% CI] | p.value | OR [95% CI] | p.value | OR [95% CI] | p.value |
| District^1^ | 0.246 [0.108; 0.561] | **0.001** | 0.104 [0.037; 0.291] | **<0.001** | 0.073 [0.025; 0.214] | **<0.001** |
| Male sex | 1.458 [0.981; 2.168] | 0.062 | 0.899 [0.669; 1.207] | 0.477 | 1.776 [1.337; 2.359] | **<0.001** |
| Male-headed household | 0.513 [0.331; 0.795] | **0.003** | 0.711 [0.523; 0.968] | **0.030** | 0.737 [0.551; 0.985] | **0.039** |
| Wealth score | 0.821 [0.702; 0.961] | **0.014** | 0.651 [0.552; 0.769] | **<0.001** | 0.602 [0.518; 0.699] | **<0.001** |
| Number of people in household | 0.919 [0.845; 1] | **0.050** | 0.903 [0.847; 0.963] | **0.002** | 0.858 [0.812; 0.906] | **<0.001** |
| Money not a big problem for seeking healthcare | 0.907 [0.566; 1.452] | 0.684 | 0.995 [0.692; 1.432] | 0.980 | 1.203 [0.869; 1.664] | 0.265 |
| Documented DPT2 vaccine | 1.612 [0.12; 21.635] | 0.719 |  |  |  |  |
| DPT2 vaccine (recall) | 1.869 [0.138; 25.245] | 0.638 |  |  |  |  |

^1^District: reference = Ndola

Model diagnostics:

| Diagnostic | Children 1 – 4 years old | Children 5 – 14 years | Adults |
| --- | --- | --- | --- |
| Hosmer-Lemeshow Goodness-of-fit p-value | 0.102 | 0.004 | 0.126 |
| C-value | 0.7517 | 0.7623 | 0.7839 |
| Somers’s D | 0.5034 | 0.5245 | 0.5677 |

Generalized variance inflation factors:

| Variable | Children 1 – 4 years old | Children 5 – 14 years old | Adults |
| --- | --- | --- | --- |
| District^1^ | 1.808 | 1.466 | 1.325 |
| Sex | 1.010 | 1.013 | 1.129 |
| Sex of head of household | 1.046 | 1.028 | 1.140 |
| Wealth | 1.851 | 1.472 | 1.336 |
| Number of people in household | 1.029 | 1.019 | 1.040 |
| Money a big problem for seeking healthcare | 1.049 | 1.019 | 1.013 |
| DPT2 vaccination | 1.044 | NA | NA |

We conclude that models fit children 1 – 4 years old and adults.
