# Supplementary material for: Who is missed in a community-based survey: Assessment and implications of biases due to incomplete sampling frame in a community-based serosurvey, Choma and Ndola Districts, Zambia, 2022
Source: PLOS Glob Public Health. 2024 Apr 29;4(4):e0003072. doi: 10.1371/journal.pgph.0003072 (PMC11057754; doi:10.1371/journal.pgph.0003072)
Supplement: S4 Appendix — (DOCX) [file pgph.0003072.s014.docx]

S4 Appendix. Selection probabilities of households in bootstrapping

To ensure that all households in a cluster with at least one individual in the eligible group had the same probability of being selected, we assigned probabilities that accounted for selection and non-response. These varied for households enrolled in the original study and those in missed population.

More precisely, for entries from the **original** dataset, the sampling probability was calculated as the probability of selection of the household from all households in the sampling frame of the original study in the clusters times the probability of selecting the respondent from all eligible respondents in that group of interest in the selected household (accounting for non-response). For example, suppose that in cluster A, 20 households were listed, had at least one child 1 – 4 years old, consented to participate in the survey, and said they would be available during the survey period. Of these, ten households were selected for interviews of children 1 – 4 years old, and eight were enrolled. The probability assigned to the eight households is 8 / 20 = 0.4. Then, for each household, we multiplied this probability by the selection probability of the child enrolled in the study among all eligible children in this age group in the household. An example is presented in the table below:

| Household | Number of children 1-4 years old enrolled | Number of children 1 – 4 years old in the household | Probability assigned to this individual |
| --- | --- | --- | --- |
| 1 | 1 | 1 | 0.4 * 1/1 = 0.4 |
| 2 | 1 | 2 | 0.4 * ½ = 0.2 |
| 3 | 1 | 1 | 0.4 * 1/1 = 0.4 |
| 4 | 1 | 3 | 0.4 * 1/3 = 0.12 |
| 5 | 1 | 1 | 0.4 * 1/1 = 0.4 |
| 6 | 1 | 1 | 0.4 * 1/1 = 0.1 |
| 7 | 1 | 2 | 0.4 * ½ = 0.2 |
| 8 | 1 | 1 | 0.4 * 1/1 = 0.4 |

For entries in missed population dataset, the probability of selection was calculated as the number of households enrolled in missed populations study divided by the total number of households eligible for missed populations study times the probability of selecting the respondent from all eligible respondents in that group of interest in the selected household.

The probability of selection within the cluster was standardized so that each individual had a probability equal to the sampling weight divided by the sum of sampling weights within that cluster.
