# Supplementary material for: Who is missed in a community-based survey: Assessment and implications of biases due to incomplete sampling frame in a community-based serosurvey, Choma and Ndola Districts, Zambia, 2022
Source: PLOS Glob Public Health. 2024 Apr 29;4(4):e0003072. doi: 10.1371/journal.pgph.0003072 (PMC11057754; doi:10.1371/journal.pgph.0003072)
Supplement: S5 Appendix — (DOCX) [file pgph.0003072.s015.docx]

**S5 Appendix. Covariates for predictive model for measles seropositivity**

The following covariates were included in the prediction model:

- District
- Cluster
- Sex of child
- Age
- Sex of head of the household
- BCG receipt
- MCV1 receipt
- MCV2 receipt
- DPT2 receipt
- DPT3 receipt
- Measles dose receipt via campaign
- Number of people in the household
- Caregiver age
- Caregiver sex
- SES variables: Main cooking source, main water source, owns agricultural land, has electricity, has television, has a plough, has a tractor, has a hammer mill, type of flooring, external wall material
- Primary caregiver’s education level
- Primary caregiver’s religion
- Healthcare seeking at referral-level facility
- History of fever and rash in the last two weeks
- Caregiver’s mobile phone ownership
- Distance to health facility a major barrier to healthcare seeking
- Money a major barrier to healthcare seeking
