# Supplementary material for: Who is missed in a community-based survey: Assessment and implications of biases due to incomplete sampling frame in a community-based serosurvey, Choma and Ndola Districts, Zambia, 2022
Source: PLOS Glob Public Health. 2024 Apr 29;4(4):e0003072. doi: 10.1371/journal.pgph.0003072 (PMC11057754; doi:10.1371/journal.pgph.0003072)
Supplement: Attachment — Submitted filename: Peer Review Paper_PGPH-D-23-01791.pdf [file pgph.0003072.s019.pdf]

Peer Review Paper, by Lazare M'BOUNGOU.

1. **Article title reviewed:** Who is missed in a community-based survey: Assessment and implications of biases due to missed populations in Zambia community-based serosurvey.

2. **Outputs of the review:**

1. The number of households selected randomly in each cluster was 40 (Within each selected cluster, all missing households were eligible if the number of missing households was less than 40; otherwise, 40 households were randomly selected): How was this number determined? Why not 30, 45, 50, ...? A brief explanation should be included in the final version).

2. The missed population study collection was conducted by a subset of data collection personnel involved in the original study: What was the advantage of using data collection staff involved in the original study? Minimization of the risk of interviewer bias? Why not a new data collection staff or a mixed data collection team? A brief explanation of your choice precisely on mitigating/controlling the risk of interviewer bias is desirable.

3. Regarding individuals agreed to participate to the survey, data collection teams conducted interviews using a shortened data collection tool from the original study: Was the data collection tool used in the original study: standard? adapted or developed? In all cases, how the content of the shortened data collection tool was assessed acceptable? A brief description should be provided in the final version of the document.

4. The investigation was carried out according to methods recognized as being rigorous based on proven mathematical theories: What softwares were used for data processing? The same as for the initial study? Please specify in the Method section.

Recommendation: For each of the four questions above, please provide a brief explanation/description/justification in the final version of the paper (Method and/or Discussion section).
