# Supplementary material for: Who is missed in a community-based survey: Assessment and implications of biases due to incomplete sampling frame in a community-based serosurvey, Choma and Ndola Districts, Zambia, 2022
Source: PLOS Glob Public Health. 2024 Apr 29;4(4):e0003072. doi: 10.1371/journal.pgph.0003072 (PMC11057754; doi:10.1371/journal.pgph.0003072)
Supplement: Attachment — Submitted filename: Response to reviewers.docx [file pgph.0003072.s016.docx]

Response Letter

We are grateful to the reviewers for their time reviewing the manuscript and for their helpful feedback and suggestions. We revised the manuscript to incorporate these suggestions. The following changes were made:

- We provided clarification on some of the prior studies carried out in Zambia. We added alternative sampling strategies that can be considered when household listing is not feasible, and we hope to have clarified the motivation behind our study.
- We also simplified the tables, removing results from multivariable analysis from tables presented in the main manuscript (although still available in the appendix), and splitting Table 2 into two tables, one for each type of caregivers.

Reviewer #1: The authors of this manuscript attempt to assess the extent to which sampling bias could affect the estimates of prevalence of measles immunity in community samples. However, their own study appear to suffer from the very own criticisms of sampling bias that they claim to address. The methodological limitations of this study are obvious from the onset. First, they characterize the ZAMPHIA sample as a "convenience sample". That is an inaccurate statement and raise serious concerns over the authors understanding of the complex sampling procedures employed in ZAMPHIA. The sample of ZAMPHIA was generated using a "stratified multistage probability sample design, with strata defined by the 10 provinces of Zambia, first-stage sampling units defined by enumeration areas (EAs) within strata, second-stage sampling units defined by households within EAs, and finally eligible persons 0-59 years of age within households."(<https://phia-data.icap.columbia.edu/storage/Country/26-10-2021-16-38-29-61782f050999c.pdf>). To call the ZAMPHIA sample a "convenience sample" is a gross misunderstanding of sampling procedures.

Response: We fully agree with the reviewer that ZAMPHIA is not an example of a convenience sample. We rephrased the introduction to make this clearer. We were referring to a study carried out on *residual samples* that were selected from the biorepository resulting from ZAMPHIA (1) and did not intend to imply that the ZAMPHIA sampling strategy was a convenience sample. While the methodology used in ZAMPHIA was a multi-stage complex sampling procedure, the study of measles seroprevalence was conducted using residual sampling from these specimens. We can assure the reviewer that we are cognizant of the efforts and the enormous scope of undertaking that studies like ZAMPHIA require, with several of co-authors involved in that study. We rephrased the section to make this clearer.

Second, the study makes no mention of the Zambia Demographic and Health Survey (DHS) 2018 (<https://dhsprogram.com/pubs/pdf/FR361/FR361.pdf>), another state-of-the-science community-based survey employing complex sampling strategies. Third, there is not a single mention of the World Health Organization Expanded Program for Immunization (WHO-EPI) random walk sampling method, nor the compact segment sampling method (<https://academic.oup.com/ije/article/33/3/469/716634?login=false>). The omission of the Zambia DHS, and a review of its sampling methods, as well as an omission of the WHO-EPI and compact segment sampling methods show that the authors did not even review the literature on sampling for epidemiological studies appropriately.

Response: We concur with the reviewer that there are a myriad of other sampling procedures. Nevertheless, our focus was on sampling strategies used for serological surveys carried out in Zambia. The 2018 Zambia DHS, as well as all its predecessors, did *not* collect biospecimens for measles serology, and was not used to estimate measles seroprevalence in Zambia. This is the reason why neither the DHS nor the WHO-EPI random walk, nor the compact segment sampling approach were cited in the overview of serological studies. While it is true that a more thorough discussion of sampling methodologies is warranted if we were solely focused on sampling in general, but since our study was necessarily nested within a study focused on serology we made these comparisons the primary point.

We recognize that the sampling approaches such as the random walk, or using geospatial sampling, provide alternative approaches to establish sampling frames that do not require listing households. Our study relied on listing because we wanted to establish sampling frames for households that had respondents in three categories of interest (children 1 – 4 years old, children 5 – 14 years old, adults). However, for studies that do not require this information, other approaches can be used, as suggested by the reviewer. We recognize that these alternatives are also subject to bias.

We incorporated a section presenting a few of these strategies as alternatives in the discussion section, and we thank the reviewer for their comment. However, a comprehensive review of diverse sampling approaches is outside the scope of this study. We included references in the discussion section to several reviews that can guide readers who are interested in learning more.

If anything, the sample used by this study is much more a convenience sample that the one obtained by ZAMPHIA, the DHS, or any sampling using the WHO-EPI or the compact segment methods. The authors use a sample that only included two districts out of 116 districts in Zambia and provide no explanation as to the sampling procedures used to obtain the sample included in this study. Consequently, the findings of this study can only be expect to refer to the inadequacies of the original, poorly designed, study that they based their research on.

Response: Our study was limited to two districts due to resource constraints; however, we disagree that this implies our results are not relevant. In fact, our study relied heavily on the complex sampling approached used by Zambia DHS, and the sampling design was led by colleagues from the Zambia Statistics Agency who were involved in roll out of both the 2018 and the 2022 Zambia DHS. We made the best attempt to replicate the DHS methodology, first using probability proportional to size to select clusters within each of the two districts (using the same Enumeration Areas as had been used in 2018 Zambia DHS). In the 2018 Zambia DHS, the teams carried out listing, from which households were selected using systematic sampling. In our study, teams carried out same approach of listing, lasting two days, and then sampled households from three separate sampling frames (one for households with children 1- 4 years old, one for households with children 5 – 14 years old, and one for households with adults (all households)). This similarity in the sampling approach with Zambia DHS was established precisely to ensure quality and representativeness of our study, despite the more limited geographic scope.

The two districts (Choma and Ndola) were selected because they captured different population profiles (primarily urban vs primarily rural). In addition, at the time of implementation of the original serosurvey, there was an ongoing serological study in three hospitals in Choma and Ndola districts using specimens from laboratory clients in these hospitals. Setting the survey in Choma and Ndola allowed for comparison of seroprevalence obtained from a community-based survey and these residual specimens (study ongoing, unpublished at this time). We added a short summary of this in the Materials and Methods section of the manuscript. In particular, we expanded the sampling procedure in the *Originally enrolled households* section of Materials and Methods.

Reviewer #2: The main question addressed by this research is very relevant and interesting. As part of efforts to quantify measles immunity gaps in Zambia, a community-based serological survey was conducted in two districts. This Community-based serological studies is well written with clear context, easy to read with the presentations and conclusions drawn consistent with arguments and evidence presented by the authors. The figures and tables aid better understanding of the work done. This research is comprehensive especially in terms of the presented content, methodology, ethical considerations and originality.
The abstract is however too long and should be shortened.

Response: We thank the reviewer for their overall response and have also updated the abstract to improve clarity and reduce the length.

The authors may wish to throw more light on rational for choosing measles and why this serosurvey was conducted in Choma and Ndola Districts ( two districts) in Zambia.

Response: We added information in the Introduction section on why we focused on measles (persistent immunity gaps in measles and setbacks in progress towards elimination both globally and in Zambia). We also highlighted that while Zambia has had great success with reaching high coverage with first dose of measles-containing vaccine, coverage with the second dose of measles-containing vaccine has lagged, and the country has been experiencing outbreaks of measles since 2022. Identifying areas with persistent immunity gaps in this context can help tailor vaccination strategies.

The two districts were selected because they captured different population profiles (primarily urban vs primarily rural). In addition, at the time of implementation of the original serosurvey, there was an ongoing serological study in three hospitals in Choma and Ndola districts using specimens from laboratory clients in these hospitals. Setting the survey in Choma and Ndola allowed for comparison of seroprevalence obtained from a community-based survey and these residual specimens (study ongoing, unpublished at this time). We added a short summary of this in the Materials and Methods section of the manuscript.

Reviewer #3: This is an excellent manuscript which is technically sound. Consistency has been maintained throughout its aim, research questions, methods, results, discussion and conclusions. The methodology has been clearly described and ethical permissions were sought from appropriate authorities. Data are presented using graphs and tables and conclusions are drawn base of the data.

Response: Thank you.

Appropriate statistical tools were used. Though the tables with statistical analysis may not be useful for lay readers. It may be worth discussing with a statisticians to keep the tables with high level data which may be more understandable to wider readers. The original tables can be included in the supplementary documents.

Response: We agree that the original tables were too cumbersome and provided too much information. We removed results from the multivariable model from the manuscript (now available in S3 Appendix). Instead, we only presented the descriptive results for healthcare seeking, separating Table 2 into two tables (one for caregivers of children 1 – 4 years old, and one for caregivers of children 5- 14 years old). We believe this makes the tables easier to read.

There is an explicit mention of the data sharing restrictions in the 'Data Availability' section and how the corresponding author can coordinate a request to the Ministry of Health of Zambia to access those data.

The language used in the manuscript is of high standard in a clear, correct, and unambiguous fashion. Any typographical or grammatical errors should be corrected at revision, so please note any specific errors here. Some minor typos are present that need to be corrected by spell-check tools. Some of these typos are as follows:

Thank you for drawing our attention to these typos. We have made the following corrections:

Line 33: magnitude of (missing) - Done
34: made not make; in not on - Done
37: distributions - Done
41: reducing – We revised the abstract for clarity and replaced the wording
56: The (missing) key - Done
71: have not has – Replaced “no World Health Organization (WHO) regions has” to “no World Health Organization (WHO) region has”
77: execution (no comma) and the (missing) - We used Oxford commas throughout but welcome guidance from the editor if this is not allowable.
104: age (no hyphen) range - Done
122: household (no comma) - We used Oxford commas throughout but welcome guidance from the editor if this is not allowable.
125: two days - Done
126: information (no comma) ...... who (not that) knew – We used Oxford commas throughout but welcome guidance from the editor if this is not allowable. We addressed the second component as suggested.

141: to (should be removed) - Done
164: were (not was) - Done
170: population - Done
260: studies – We believe this should be “study,” as it refers to a single missed population study
316: hospitals- Done
458: a (missing) sub-national - Done
