# Supplementary material for: Who is missed in a community-based survey: Assessment and implications of biases due to incomplete sampling frame in a community-based serosurvey, Choma and Ndola Districts, Zambia, 2022
Source: PLOS Glob Public Health. 2024 Apr 29;4(4):e0003072. doi: 10.1371/journal.pgph.0003072 (PMC11057754; doi:10.1371/journal.pgph.0003072)
Supplement: Attachment — Submitted filename: Report to PGPH Z.docx [file pgph.0003072.s018.docx]

Dear Editor,

Thanks for the opportunity to review this manuscript. My recommendations are below.

1) The title should meet the SMART requirements.
2) In the Abstract part it should include the sample size, sampling techniques, study period and area
3) In the introduction part, start your reference by cited number 1, not merged like 1_4 2. you have to mentioned rationale of your study at end of the introduction
4) Operational definition for missed and original population
5) You have to write your all tables Full title including year, place and description in the document
6) Is that DPT or Pentavalent vaccines strategies implemented in Zambia?
7) In conclusion part, You have to summarize your conclusion based on findings
8) What about your recommendations based on findings
9) References should be up to date, old references example reference number 4 and 19.

Best regards
