# Supplementary material for: Who is missed in a community-based survey: Assessment and implications of biases due to incomplete sampling frame in a community-based serosurvey, Choma and Ndola Districts, Zambia, 2022
Source: PLOS Glob Public Health. 2024 Apr 29;4(4):e0003072. doi: 10.1371/journal.pgph.0003072 (PMC11057754; doi:10.1371/journal.pgph.0003072)
Supplement: Attachment — Submitted filename: Response to reviewers.docx [file pgph.0003072.s020.docx]

Response Letter

We are grateful to the reviewers for their time reviewing the manuscript and for their helpful feedback and suggestions. We revised the manuscript to incorporate these suggestions. Notably, we provided more explanations about methodological choices, such as restricting the number of households per cluster in the missed populations study and use of a subset of study team that carried out the original survey. We updated the manuscript title, abstract, and table titles to be more specific and comprehensive. We provided operational definitions of missed and original populations and edited the conclusion to be more directly linked to the findings.

We also provided an updated contact for the Acting Director of the Zambian National Health Regulatory Authority in the data availability statement, following change in leadership.

We thank the reviewers for their support in improving this manuscript.

Reviewer #4:

Comment: The title should meet the SMART requirements.

Response: We updated the title to be better meet the SMART requirements. It now reads as follows: “Who is missed in a community-based survey: Assessment and implications of biases due to incomplete sampling frame in a community-based serosurvey, Choma and Ndola Districts, Zambia, 2022”.

Comment: In the Abstract part it should include the sample size, sampling techniques, study period and area

Response: We have incorporated information about sample size, sampling approach (stratified multi-stage cluster survey for the original serosurvey, and stratified survey for missed populations follow-up), study period (May – June 2022 for original serosurvey, and July – August 2022 for missed populations follow-up), and the two districts where the survey took place (Ndola and Choma Districts). The changes are reflected in the abstract.

We also revised the abstract by removing some information about the threshold approach to remain within the 300 word limit.

Comment: In the introduction part, start your reference by cited number 1, not merged like 1_4 2. you have to mentioned rationale of your study at end of the introduction

Response: We updated the references as suggested. We also included a more specific rationale, situated within the larger objective of wanting to understand the extent to which sampling bias may be present in the serosurvey. Specifically, the text now reads, “To understand the extent to which sampling bias may be present and affect the results of a community-based serosurvey conducted in two districts of Zambia in 2022, we conducted a follow-up study of households missed from the serosurvey’s sampling frame. Specifically, we wanted to understand whether the two populations (those included in the original serosurvey and those missed from its sampling frame) differed by some characteristics. Further, we wanted to evaluate whether the exclusion of these households was likely to result in biased estimates of the outcomes of interest.”

Comment: Operational definition for missed and original population

Response: We added an operational definition in the Method section. Namely, original” population refers to the study sample obtained from the serosurvey carried out in April – June 2022. “Missed” population refers to the study sample included in the follow-up survey carried out in July – August 2022. These were households selected among those eligible for the original serosurvey but not listed or reported to be unavailable during the study period.

Comment: You have to write your all tables Full title including year, place and description in the document

Response: We updated the table and figure titles to include this information.

Comment: Is that DPT or Pentavalent vaccines strategies implemented in Zambia?

Response: Pentavalent vaccines are used in Zambia. We updated the text as well as the figure to reflect this.

Comment: In conclusion part, You have to summarize your conclusion based on findings

Response: We updated the conclusion to build upon the findings (namely, the differences between the two populations, including household size, occupation, wealth, and healthcare seeking). We also specified that simulations resulted in <5% estimated bias in outcomes of interest. We believe this better captures the study findings.

Comment: What about your recommendations based on findings

Response: We updated the conclusion to make an explicit recommendations for making additional efforts to obtain a more comprehensive sampling frame.

Comment: References should be up to date, old references example reference number 4 and 19.

Response: We replaced the older reference (Ref. 4) providing an example of the use of serology as applied to tetanus with newer references. We would like to keep Ref. 19, even though it is older, as it provides a well conducted study showing comparability of estimates from a random cluster survey and residual samples from laboratories and believe that the findings are relevant despite their age. However, we added two newer references, one focusing on estimates from blood donors and general population, and one looking at cluster random sampling and a mapping approach using a school-based sample for estimating prevalence of trachomatous inflammation–follicular.

Reviewer #5:

Comment: The number of households selected randomly in each cluster was 40 (Within

each selected cluster, all missing households were eligible if the number of

missing households was less than 40; otherwise, 40 households were randomly

selected): How was this number determined? Why not 30, 45, 50, …? A brief

explanation should be included in the final version).

Response: We selected 40 as a cut-off mostly for operational purposes, as restricting the number of sampled households per cluster to 40 allowed us to reach the desired sample size in both districts while spending on average a week in each cluster and maintaining a reasonable workload per team. We also did not think that going beyond 40 households would be effective in obtaining additional information about the missed households. We included a brief explanation of this in the Methods.

Comment: The missed population study collection was conducted by a subset of data

collection personnel involved in the original study: What was the advantage of

using data collection staff involved in the original study? Minimization of the

risk of interviewer bias? Why not a new data collection staff or a mixed data

collection team? A brief explanation of your choice precisely on mitigating/controlling the risk of interviewer bias is desirable.

Response: Yes, exactly! Minimizing interviewer bias was one of the reasons we chose to use a subset of the original data collection team. We also wanted to capitalize on familiarity with the questionnaire. Another advantage of using the same team was familiarity with the clusters. Finding missed households was not an easy task, especially when GPS coordinates were not precise, or details about the location of missed households were vague (for example, if a data collector noted in the information team that the missed household was located “near the church”). Using the same teams for data collection simplified the task of finding the households. Finally, the teams had a better idea of the challenges of working in these areas and the expectations that we had for them, including road conditions, hills, and heat. We anticipated that the missed populations follow-up study would be even more challenging logistically and felt that using a subset of the study team would be an advantage. We added some of this information in the Methods section.

Comment: Regarding individuals agreed to participate to the survey, data collection teams conducted interviews using a shortened data collection tool from the original

study: Was the data collection tool used in the original study: standard? adapted

or developed? In all cases, how the content of the shortened data collection tool

was assessed acceptable? A brief description should be provided in the final

version of the document.

Response: We tried to use standard questions as much as possible to make sure the questions were valid, acceptable, and would allow for comparison across surveys. We relied heavily on the 2018 Zambia Demographics and Health Survey (DHS) and were able to use many of the same demographic questions as well as some on healthcare seeking and vaccination from the DHS. We also used some of the questions from the Zambia Living Conditions Monitoring Survey. However, we adapted and developed several other questions. For example, we wanted to understand specifically healthcare seeking at the district-level hospitals and this question was developed specifically for the questionnaire. The questionnaires were piloted during data collection team training.

For the shortened data collection tool used in the missed populations follow-up survey, we did not include any new questions. However, we dropped certain modules that were not of interest for this study, namely the travel survey module, and we did not collect blood samples or temperature of participants. We found that in the serosurvey most of the participants consented to participate in the questionnaire and completed the interviews, which highlighted the high acceptability of the questionnaire. However, there was some refusal of blood draws. Based on this information, we deemed that the shortened questionnaire, with no blood draws, would be highly acceptable to missed populations participants. This was indeed the case.

We provided this information in the Methods section of the revised manuscript.

Comment: The investigation was carried out according to methods recognized as being

rigorous based on proven mathematical theories: What softwares were used for

data processing? The same as for the initial study? Please specify in the Method

section.

Response: We used CSPro to collect data for both the serosurvey and the missed populations follow-up study. We used R and Rstudio for all data processing and analyses. We added this information, including citations for the software, in the Methods section of revised manuscript.
