# Supplementary material for: Who is missed in a community-based survey: Assessment and implications of biases due to incomplete sampling frame in a community-based serosurvey, Choma and Ndola Districts, Zambia, 2022
Source: PLOS Glob Public Health. 2024 Apr 29;4(4):e0003072. doi: 10.1371/journal.pgph.0003072 (PMC11057754; doi:10.1371/journal.pgph.0003072)
Supplement: Attachment — Submitted filename: review article Sarashwati Giri.docx [file pgph.0003072.s021.docx]

# Recommendations for Methodology Section:

Here are some comments and suggestions:

The methodology section is comprehensive, but for improved clarity, consider breaking down lengthy paragraphs, incorporating visual aids, and providing more details on the classification of missed households and the sampling process.

Elaborate on the development of data collection tools and provide context for the chosen variables in population comparisons.

Additionally, clarify the rationale behind the mixed-effects model and offer a brief overview of the simulation analysis. Proofreading for grammatical errors will enhance overall readability.

# Operational Definition Section:

1. Clearly Define Missed Populations: Provide a concise and clear operational definition of "missed households" and how they were identified, ensuring readers understand the criteria used for exclusion.

2. Justification for Selected Power Parameters: Briefly explain the rationale behind choosing an 80% power and alpha = 0.1 for the missed population survey, especially in the context of the assumed vaccination coverage of 90%.

# Results

- The part where they talk about the results is well-organized and easy to understand. They used the right statistical tests to check different things and even included some numbers to show how strong the connections are between different factors.
- They kept things consistent when talking about results from different areas and groups, which helps make everything clear.
- They went beyond just giving basic numbers and explained more about how different factors are connected. They also talked about things like wealth and where people live, which gives more context to the findings.
- They were careful in checking if their statistical models were good, showing they really thought about how reliable their results are.
- They didn't just present good things – they also talked about the limitations of their study, like where their model didn't fit perfectly and possible biases in their results.
- In a nutshell, the results part of the study is well done. It tells us a lot, and the researchers were open about the study's strengths and weaknesses.

# Discussion

The discussion section is well-structured and thoughtfully analyzes the study's findings. It adeptly interprets the potential biases, addressing demographic differences between included and missed households, contributing to a distinct understanding of the study outcomes. By considering the generalizability of results and engaging with methodological strengths and limitations, it adds valuable insights to the broader research discourse. Drawing comparisons with other studies and offering recommendations for future research enhances the study's relevance. The reflection on social desirability bias demonstrates a thorough exploration of potential sources of bias, and the acknowledgment of limitations adds to the study's credibility. Overall, the discussion provides a comprehensive and insightful reflection on the research outcomes.
